# Adverse reactions to the injection of face and neck aesthetic filling materials: a systematic review

**DOI:** 10.4317/medoral.25713

**Published:** 2022-12-24

**Authors:** Renato Assis Machado, Lilianny Querino Rocha de Oliveira, Hercílio Martelli-Júnior, Fábio Ramoa Pires, Janaína Badin Carvas, Victor Edson Rogerio, Viviane de Azevedo Rabelo, Ricardo D Coletta

**Affiliations:** 1 Department of Oral Diagnosis, School of Dentistry, University of Campinas (FOP/UNICAMP), Piracicaba, São Paulo, Brazil; 2 Hospital for Rehabilitation of Craniofacial Anomalies, University of São Paulo, Bauru, São Paulo, Brazil; 3 Graduate Program in Oral Biology, School of Dentistry, University of Campinas, Piracicaba, São Paulo, Brazil; 4 Stomatology Clinic, Dental School, State University of Montes Claros (UNIMONTES), Montes Claros, Minas Gerais, Brazil; 5 Center for Rehabilitation of Craniofacial Anomalies, Dental School, University of José Rosario Vellano (UNIFENAS), Alfenas, Minas Gerais, Brazil; 6 Oral Pathology, Dental School, Rio de Janeiro State University, Rio de Janeiro, Brazil; Post-Graduation program in Dentistry, Estácio de Sá University, Rio de Janeiro, Brazil; 7 Orofacial Institute of the Americas (IOA), São Paulo, Brazil

## Abstract

**Background:**

Adverse reactions, caused during the inflammation and healing process, or even later, can be induced by the injection of dermal filler and can present a variety of clinical and histological characteristics. In this study we aimed to review the adverse reactions associated with the injection of aesthetic filling materials in the face and neck.

**Material and Methods:**

The review was reported according to the Preferred Reporting Items for Systematic Reviews and Meta-Analyses checklist. Studies published that mentioned adverse reactions in patients with aesthetic filling materials in the face or neck were included. Risk of bias was assessed using the Joanna Briggs Institute appraisal tool. After a 2-step selection process, 74 studies were included: 51 case reports, 18 serial cases, and five cohorts.

**Results:**

A total of 303 patients from 20 countries were assessed. Lesions were more prevalent in the lip (18%), nasolabial folds (13%), cheeks (13%), chin (10%), submental (8%), glabella (7%), and forehead (6%). Histopathological analysis revealed a foreign body granuloma in 87.1% of the patients, 3% inflammatory granuloma, 3% lipogranuloma, 2.3% xanthelasma-like reaction, 1% fibrotic reaction, 0.7% amorphous tissues, 0.7% xanthelasma, 0.3% sclerosing lipogranuloma, 0.3% siliconoma, and 0.3% foreign body granuloma with scleromyxedema. In addition, two patients displayed keratoacanthoma and two others displayed sarcoidosis after cutaneous filling. The most commonly used materials were silicone fillers (19.7%), hyaluronic acid (15.5%), and hydroxyethyl methacrylate/ethyl methacrylate suspended in hyaluronic acid acrylic hydrogel (5.6%). All patients were treated, and only 12 had prolonged complications.

**Conclusions:**

There is evidence that adverse reaction can be caused by different fillers in specific sites on the face. Although foreign body granuloma was the most common, other adverse lesions were diagnosed, exacerbating systemic diseases. In this way, we reinforce the importance of previous systemic evaluations and histopathological analyses for the correct diagnosis of lesions.

** Key words:**Facial filler, reaction lesion, granulomatous reaction, face.

## Introduction

Collagen, the main component of the dermis, contributes to skin strengthening and support. As an individual’s age increases, the physiological activity of fibroblasts deteriorates, resulting in decreased tissue volume and elasticity (1). Currently, to compensate for these cosmetic facial deformities, the injection of facial dermal filler materials is at an all-time high. Several different health professionals, including dentists, biomedical scientists, and pharmacists, apart from doctors, are able to perform these procedures.

Over the years, several materials have been used to correct signs of aging in the face or neck, including permanent and non-permanent dermal fillers, implants, neurotoxins, lasers and micropigmentation, without actual determination of which material is the ideal one (2,3). Thus, materials for facial fillers can be classified into two broad categories: biological substances (e.g., collagen or adipose tissue) and non-biological substances (e.g., silicone oil or agarose). Biological substances are derived from animal and non-animal sources. Animal substances can be obtained from the same person (autologous), another person (usually a cadaver [homologous]), or another animal (heterologous). They can also be synthetically manufactured. Non-animal biological substances can be obtained from non-animal organisms (e.g., dextran granules derived from bacteria). Non-biological substances can be obtained from petroleum (e.g., polytetrafluoroethylene) or minerals (e.g., silicone), or they can be re-synthesized (3).

After dermal filler, the immediate effects to up to 15 days effects are usually swelling, pain, erythema, itching, bruising at the application site, hypersensitive reaction, infection (herpes simplex virus, bacterial abscess/cellulitis), Tyndall effect, superficial irregularities and nodules, occlusion vascular disease, local tissue necrosis, and blood vessel embolization (blindness, stroke) (4). However, adverse reactions are also reported, ranging from weeks to years after the filling procedure (5,6). Thus, we conducted a systematic review to analyze all cases of histologically diagnosed adverse reactions to aesthetic fillers in the face or neck (HDARAFFN).

## Material and Methods

- Protocol

This systematic review was developed according to the Preferred Reporting Items for Systematic Reviews and Meta-analyses (PRISMA) checklist (7,8), and included a literature search strategy, selection of articles through inclusion and exclusion criteria, data extraction, and quality assessment.

- Study Design and Eligibility Criteria

This systematic review investigated HDARAFFN. The literature search was conducted without time. The patient, intervention, comparison, outcome (PICOS) strategy was used to construct the research question with the following inclusion criteria: (i) population: patients undergoing filling materials to rejuvenate their face or neck; (ii) intervention: clinical and histopathological evaluation to identify adverse reaction; (iii) comparison: none; (iv) outcome: frequency and location of adverse reaction; and (v) study design: observational studies (case reports, serial cases, and cohort studies).

Studies were excluded using the following criteria: (i) studies evaluating filling materials in areas of the body other than the face or neck; (ii) studies without histopathological analyses; and (iii) reviews, letters, personal opinions, book chapters, and conference abstracts.

- Information Sources and Search Strategy

Electronic search strategies for the following bibliographic databases were developed (Supplement 1): Cochrane (https://www.cochranelibrary.com), Embase (https://www.embase.com), Livivo (https://www.livivo.de), PubMed (https://pubmed.ncbi.nlm.nih.gov), Scopus (https://www.scopus.com), and Web of Science (https://www.webofscience.com/wos/woscc/basic-search). An additional search in gray literature (ProQuest) was performed, as well as a manual search of the reference lists of the included studies. The search included all articles published up to July 12, 2022, across all databases. Rayyan software reference manager was used to collect references and remove duplicate articles. The same search strategy was used for every update.

- Study Selection

Study selection was completed in two phases. In Phase 1, two authors (R.A.M. and L.Q.R.O.) independently reviewed the titles and abstracts of all references using Rayyan software (9), selecting those that met the inclusion criteria, and discarding the remaining studies. The third author (H.M.J.) was involved when required to make a final decision on the inclusion or exclusion of a study. In Phase 2, the same selection criteria were applied to full-text articles to confirm the studies that reported HDARAFFN. The same two authors independently participated in Phase 2. The reference lists for all included articles were critically assessed by the three authors, and new articles were selected for selection analysis. Any disagreement in either phase was resolved through discussion and mutual agreement between the three authors. The final selection was based on full-text articles.

- Data Collection

Initially, the first (R.A.M.) and second (L.Q.R.O.) authors collected the required information from the selected articles. Then, the third author (H.M.J.) cross-checked the collected data and confirmed its accuracy. Any disagreement was resolved through discussion and mutual agreement between the three authors. Experts were involved as required for the final decision. When the necessary data could not be retrieved, attempts were made to contact the article authors to retrieve the missing information.

- Risk of Bias Within Studies

Risk of bias was independently evaluated by two authors (R.A.M. and L.Q.R.O.) using the Joanna Briggs Institute’s Critical Appraisal Checklist for Studies Reporting Prevalence Data and the Critical Appraisal Checklist for Case Reports (10). The third author (H.M.J.) was consulted for any case of disagreement. Decisions about scoring were discussed by all authors before the critical appraisal assessments, and a study was characterized as having a high risk of bias when it reached a “yes” score of up to 49%, moderate when it reached 50% to 69%, and low when it was >70%.

## Results

- Study selection and characteristics

**Figure 1:**
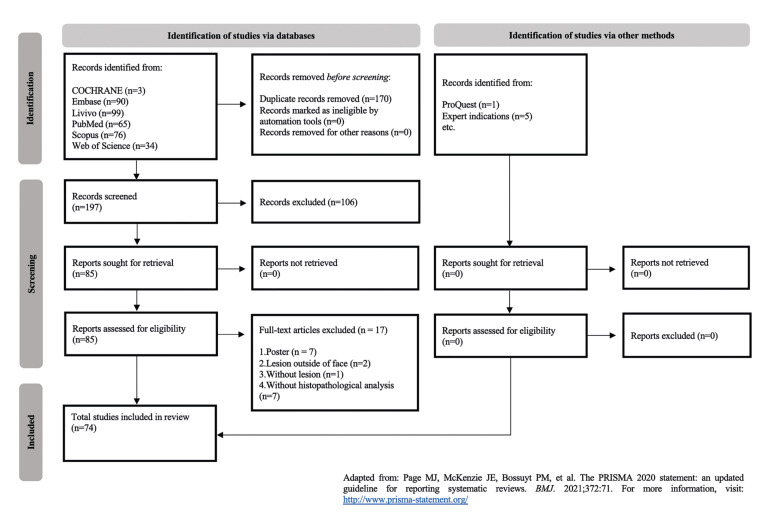
Flow diagram of literature search and selection criteria adapted from PRISMA.

In the first phase, 367 records were identified from the explored databases, and after removing the duplicates, 197 remained for title and abstract screening. After all records were evaluated, 91 articles were selected for the second phase. Then, full-text reading was conducted, and 17 studies were excluded due to the predefined eligibility criteria. Next, 74 studies were selected for synthesis (Supplement 2), all of which had a HDARAFFN. A flowchart detailing this process is shown in Fig. 1.

The included studies were conducted in differing parts of the world, with 47.3% performed in Europe, 24.3% in North America, 21.6% in Asia, and 6.8% in South America (Supplement 3). All studies were published between June 2001 and October 2021, and were written in English.

- Synthesis of Studies

A total of 303 patients were included in this systematic review. Two hundred and fourteen were women, 10 men, and 79 were not reported as males or females. The mean age was 51.9 years old (13.0). All patients had a face or neck filling: 60 (19.7%) used silicone, 47 (15.5%) hyaluronic acid, 17 (5.6%) hydroxyethyl methacrylate/ethyl methacrylate suspended in hyaluronic acid acrylic hydrogel (Dermalive®), 17 (5.6%) calcium hydroxylapatite, 16 (5.3%) polyalkamide, 15 (5.0%) poly L-lactic acid, 15 (5.0%) autologous fat, eight (2.6%) polyacrylamide gel, six (2.0%) polymethylmethacrylate, five (1.6%) collagen, three (1.0%) alginate, three (1.0%) polyalkylimide, three (1.0%) polycaprolactone, two (0.7%) acrylic hydrogel particles, and one (0.3%) methacrylate gel. Multiple fillers were used in 37 (12.2%) patients and in 49 (16.1%) patients’ fillers were unknown (Supplement 3).

An evolution time of 10 days to 40 years was observed for the appearance of lesions after aesthetic filling, and most lesions were on the lip (*n*=79), followed by nasolabial folds (*n*=58), cheeks (*n*=56), chin (*n*=41), submental (*n*=33), glabella (*n*=29), forehead (*n*=25), tear trough/infraorbital (*n*=22), nose (*n*=19), periorbital (*n*=16), zygomatic arches/malar (*n*=12), temples (*n*=10), perioral (*n*=7), mentolabial folds (*n*=6), lid (*n*=4), jaw (*n*=3), maxillary vestibule (*n*=1), and neck (*n*=1). Three patients had lesions at multiple sites, and three patients’ lesion sites were not described (Table 1).

**Table 1:**
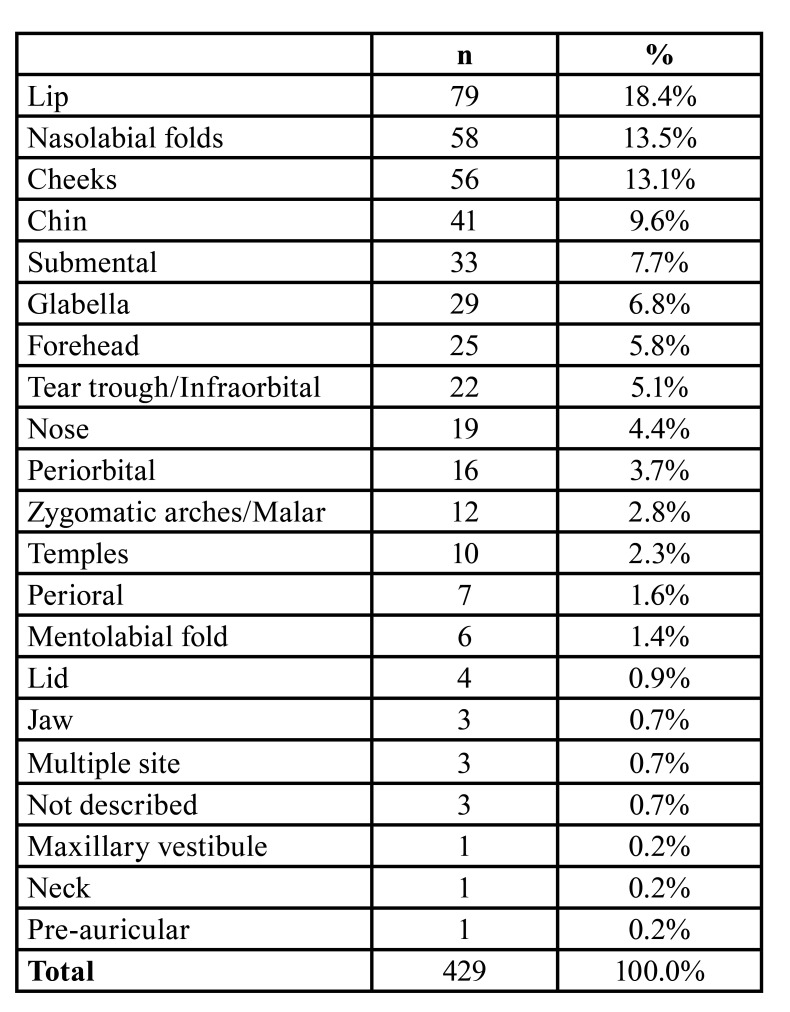
Anatomical location of the facial or neck adverse reaction to aesthetic filling.

Biopsy with histological examination was performed in all cases, and the most common lesion was a foreign body granuloma (*n*=264). However, the patients were also diagnosed with other lesions. Nine patients had inflammatory granuloma, nine lipogranuloma, seven xanthelasma-like reactions, three fibrotic reactions, two amorphous tissues, two xanthelasmas, two keratoacanthomas, two sarcoidoses, one sclerosing lipogranuloma, one siliconoma, and one foreign body granuloma with scleromyxedema (Table 2). Although all lesions were treated, the filler materials used and the relevant clinical conditions differed. The lesion treatments varied with follow-up from six days to eight years. Only 12 patients had treatment complications (Supplement 4).

**Table 2:**
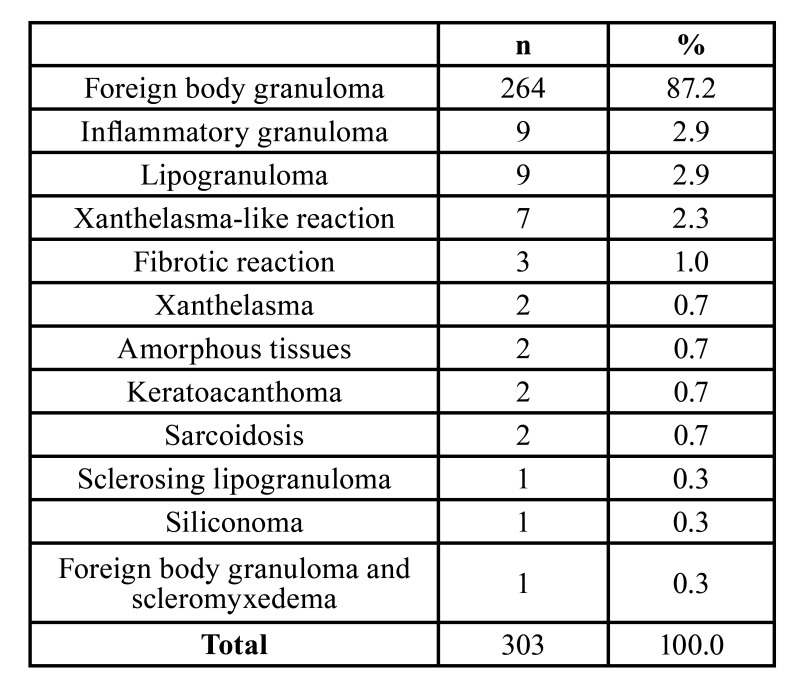
Histological pattern of the adverse reaction in face and neck after aesthetic filling.

- Risk of Bias Within Studies

Results of the risk of bias assessments for individual studies are described in Supplement 5. Case reports and cross-sectional studies were evaluated with the appropriate checklist for each study design (10).

## Discussion

The approach used in this systematic review was chosen to identify all studies to date that have HDARAFFN. The findings from 303 patients showed that 264 (87.1%) had foreign body granuloma. Histopathologically, the dermal filler foreign body reaction shows dense lymphohistiocytic infiltration with eosinophils, and granulomatous infiltrates with foreign body giant cells (11). However, findings of mild lymphohistiocytic infiltrate without granulomas are common, with some reports showing moderate lymphocytic and plasma cell infiltrates without foreign material or foreign body reactions (12,13). Of all patients, only nine were diagnosed with inflammatory granuloma and six were diagnosed with amorphous tissue, fibrotic reaction, or an association of foreign body granuloma with scleromyxedema after acrylic hydrogel particles, biopastique, collagen, hyaluronic acid, polyacrylamide, or polyalkylimide gel.

Non-immunologic granulomas, such as foreign body granuloma formation due to inorganic matter (e.g., silica, silicone), can be distinguished by the absence of lymphocytes in the lesion. The histology of a typical immunological granuloma is a macrophage/epithelioid core surrounded by a cuff of lymphocytes, where considerable fibrosis may also occur (6). Multiple vacuolated cyst-like structures and “swiss cheese” appearances are also possible (11). Only one patient was diagnosed with siliconoma after unknown filler materials in the glabella, cheeks, nasolabial, and perioral areas (14).

Two women showed xanthelasma after hyaluronic acid filler (15,16) and seven cases of xanthelasma-like reaction by filling with calcium hydroxylapatite and polycaprolactone (17). Xanthelasma appears as yellowish, flat, and soft plaques located most commonly on the medial portion of the eyelid. They usually appear in women aged 30–50 years and may also present as semisolid or calcareous masses. The patients in this systematic review had a mean age of 46.5 years [±5.4]. The youngest individuals were in their first two decades of life and may have a family history of xanthelasma, with or without hyperlipidemia (18,19). The mechanisms that initiate macrophage accumulation, cholesterol uptake, and foam cell formation in normolipemic patients with no evidence of xanthelasma pre-filler injection are not clearly understood. However, it has been suggested that hyaluronic acid injections in the extracellular matrix are capable of binding extravasated low-density lipoprotein (LDL). The LDL-glycosaminoglycan complex is internalized by macrophages and histiocytes more intensely than native LDL, which may play a role in xanthelasma formation (20).

Unlike xanthelasmas, which occur only on the eyelids, lipogranulomas can occur in different areas. They exhibit locules of extracellular lipids surrounded by epithelioid histiocytes and other inflammatory cells (21). The chalazion is the prime example of a lipogranuloma, a blockage in the draining duct of a meibomian gland secretory unit with extrusion of the lipid synthesized by the meibomian acinar cells into the tarsus, surrounding orbicularis muscle and dermis. Extracellular lipids irritate and induce a granulomatous response. The histiocytes in the chalazion are mostly non-lipidized (non-xanthomatous). Other causes of lipogranulomas include trauma to the orbital fat, injections of foreign lipidic material or cosmetic fillers, infection, exuberant idiopathic inflammation predominantly involving the orbital fat, and vasculitis (21,22). In this systematic review, we found nine cases of lipogranuloma in the forehead, cheek, glabella, temporal area, and tear trough (23) and one case of sclerosing lipogranuloma in the nose (24).

Cutaneous sarcoidosis has been reported in scars, tattoo sites, venipuncture, and intramuscular and hyaluronic acid injections after a variable period of a few months to 38 years (25). Regarding the onset of sarcoidosis following the use of cutaneous fillers, it is known that the longer the time interval between the two events, the lower the possibility of it being a foreign body granuloma, which is an important differential diagnosis of cutaneous sarcoidosis (26,27). According to the literature, the application of the filler can evidence signs of subjacent sarcoidosis or even trigger the clinical picture in a previously predisposed patient (28). In this systematic review, two cases showed that the procedure was the probable triggering factor for the clinical manifestation of the systemic disease, characterized by cutaneous lesions and pulmonary involvement rather than isolated involvement of the skin after local injection, as descript by Ortiz-Álvarez *et al*. (29). The following features corroborated the diagnosis: radiological characteristics typical of pulmonary involvement; granulomatous cutaneous manifestations on the face, but not exactly where the previous application of the filler was conducted; histology compatible with sarcoidal granuloma rather than with the foreign body type or other granulomatous disease forms.

Two men developed keratoacanthoma after collagen injection, as descript by Brongo *et al*. (30). Keratoacanthoma is a common squamous neoplasm that originates in pilosebaceous glands. Although the exact cause of keratoacanthoma is unknown, an association with sun exposure, genetic factors, immunosuppression, carcinogens, viruses, and trauma has been reported. Histologically, keratoacanthoma is characterized by deep bulbous lobules of keratinizing, well-differentiated squamous epithelium with a keratin-filled central crater. There was marked acanthosis with hyperkeratosis, and little or no parakeratosis. Keratoacanthoma grows rapidly and, in most cases, resolves spontaneously. Although keratoacanthoma rarely progresses to metastatic carcinoma, its early diagnosis and treatment are recommended. In one case, excision was performed, and the other was started on oral acitretin 50 mg once daily, and had a 75% improvement in his lesions. Moreover, keratoacanthoma has been reported with other cosmetic procedures, such as CO2 laser resurfacing and body peeling (31,32).

The topography most affected by adverse reactions was the lip (18.4%), although other perioral regions (16.5%) were also affected (nasolabial folds, perioral, and mentolabial fold). In a recent systematic review, 66 lip lesions were observed after dermal fillers (33) and in this systematic review, we identified 150 cases with adverse reaction histopathologically confirmed in the lip and perioral regions. Adverse reactions were reported in other areas such as the chin/submental (17.3%), cheek/malar (16.1%), eyelids (9.7%), and glabella (6.8%), among others. Studies have shown that the eyes and mouth (due to smiles) are the most pleasing facial features and are therefore one of the most sought-after regions for aesthetic procedures (34,35). There is also an increasing global consensus across different ethnic groups and regions on the defining attributes of facial beauty. Pronounced cheekbones and a defined jawline are such attributes that are considered the ideal criteria for facial beauty (36). Although it has been reported that the area with the greatest risk of vision loss is the glabella, it is one of the areas that are most filled (37,38). In this study, we observed that the glabella is also one of the areas that presents a lot of adverse reactions.

In summary, this study provides evidence that different aesthetic materials can cause adverse reactions and exacerbate systemic diseases or neoplasms. Thus, we emphasize the value of histological assessment and preceding systemic evaluation for proper diagnosis of adverse reactions.
